# Neighborhood perceivable graph neural network for relational heterogeneous Twitter bot detection

**DOI:** 10.1371/journal.pone.0342686

**Published:** 2026-02-17

**Authors:** Yan Li, Haoyu Lu, Wanying Chen

**Affiliations:** 1 WuXi University, Wuxi, China; 2 Henan Key Laboratory of Cyberspace Situation Awareness, Zhengzhou Science and Technology Institute, Zhengzhou, China; 3 Zhengzhou Information Engineering College, Zhengzhou, China; University of Nottingham Ningbo China, CHINA

## Abstract

Malicious bots undermine the integrity and safety of online social platforms, making their detection an urgent priority. This work aims to address the limitations of existing GNN-based bot detection approaches, particularly their inability to adapt the aggregation process to local feature distributions and heterogeneous relational structures. Our proposed framework, NeighborSense, exploits both relational graph structures and node features for detecting social bots. The approach involves an analysis of local bot-human interaction patterns, leading to the development of two local metrics based on neighborhood statistics. We then use a dynamically maintained shortcut module to integrate the above two metrics into a relational graph convolutional neural network (R-GCN) learning process, enabling gated aggregation control for different users based on the feature distribution of their neighbors. We confirmed that the proposed R-GCN backbone along with the metric-based adaptive gating mechanism achieves relational heterogeneity awareness, local entropy awareness, and local feature heterogeneity awareness. Benchmarking against state-of-the-art methods reveals that NeighborSense consistently achieves higher detection accuracy.

## Introduction

According to disclosures made to the US Securities and Exchange Commission (SEC), Twitter has reported that fewer than 5% of its accounts are spam or fraudulent. However, this stands in contrast to estimates within academic research, which suggest that the proportion of bots on social media platforms is substantially larger. For instance, a study by [1] estimated that bots constitute between 9% and 15% of active Twitter users. Malicious actors create and operate such accounts to disrupt social networks through spam, artificial traffic amplification, and manipulation of public opinion, thereby introducing serious security risks. As a result, detecting social bots has emerged as a critical challenge for online social networks (OSNs).

Graph neural networks (GNNs) have achieved considerable progress in learning from structural data in recent years, leading to the rise of GNN-based techniques for social bot detection and establishing this area as a prominent research direction. By employing neural networks to iteratively gather neighborhood information, these approaches effectively incorporate both structural and attribute data from OSNs. The resulting aggregated features thus benefit from the strengths of prior feature-based techniques [2] as well as graph-based methodologies [3, 4].

As an illustrative example, the BotRGCN model [5] is implemented on a Twitter follower-followee graph using relational-graph convolutional networks, processing multi-modal user information including semantic, property, and neighborhood data. In a similar vein, the work by Feng *et al*. [6] designed a bot detection framework that leverages heterogeneous information networks (HINs) to capture the heterogeneity within Twitter’s structure.

Despite the success of GNN-based methods on social bot detection, we observe that they still have the following limitations.

The existing GNN-based bot detection methods incorporate the graph structure information to node embeddings through neighborhood aggregation. However, they do not control the strength of local aggregation effectively. Note that the current approach based on the attention mechanism only focuses on the point-to-point aggregation control based on node features, but does not incorporate the feature distribution of neighborhoods. For example, during an online opinion outbreak, the feature of a human user may be diluted by a large number of bot user attacks, which reduces the detection accuracy of that user. This limitation cannot be effectively addressed by point-to-point attention mechanisms alone. Moreover, the existing GNN models are not yet able to learn and control the aggregation strength of edges with different directions and types effectively. As in the case of neighborhood aggregation processes for heterogeneous social relations such as “followed by” and “following”, “likes” and “blocks”, the aggregation strength should obviously be differentiated both overall and locally.

**Fig 1:**
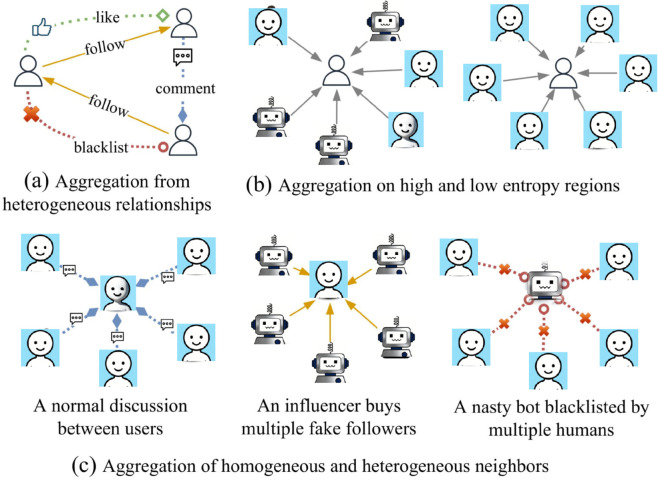
Three key issues to be addressed.

We translate the above issues into the following specific **design goals**:

- Relational heterogeneity awareness. As shown in Fig 1(a), the proposed model should be able to adapt to multiple social relations and learn aggregation strategies about different types of edges efficiently.
- Local entropy awareness. As illustrated in Fig 1(b), the aggregation strategy in our model is adjusted depending on the feature distribution within the neighborhood of the central node. This enables a clear distinction between aggregation over heterogeneous neighbors and that over homogeneous ones.
- Local feature heterogeneity awareness. Fig 1(c) illustrates three typical cases commonly found in real social networks, from which it can be seen that the aggregation strategy of the proposed model should be adjusted according to the degree of heterogeneity between the center user and its neighbors. Generally, if the learned label of a center user and its neighbors disagree, further aggregation may hurt performance, whereas when the learned feature of a center user is nearly identical to its neighbors, further aggregation is unnecessary.

**Solution and contributions:** In this paper, we propose NeighborSense, a GNN-based social bot detection method that takes advantage of both user attributes and structural information of multiple social relations. On this basis, we designed two metrics with which a dynamically updated shortcut module learns the aggregation strategies mentioned above to model more refined social interaction patterns.

In our framework, the social network is modeled as a graph with users depicted as nodes and their interactions as edges of multiple relation types. This heterogeneous graph is processed via a Relational Graph Convolutional Network (R-GCN) [7] serving as the backbone, where the aggregation mechanisms within its layers provide the foundation for node classification. We first adopt two metrics to quantify the feature distribution and homogeneity of nodes and their neighbors. Using these metrics, we propose an adaptive shortcut module that allows each node to independently aggregate neighborhood information based on its neighbors’ prior predictions. As a result, different nodes may experience different aggregations according to relational heterogeneity, local entropy, and local feature heterogeneity.

Our key contributions are:

- We investigate the local interaction patterns between bot and human accounts, and introduce two novel metrics to characterize node-level neighborhood statistics.
- We propose a GNN-based detection framework that integrates node attributes and multiple types of social relations. A dynamically updated shortcut module is designed to incorporate the proposed metrics into the graph learning process, enabling adaptive gated aggregation conditioned on the feature and label distributions of each node’s neighbors.
- Experimental results demonstrate that the proposed approach, built upon an R-GCN backbone augmented with the metric-guided gating mechanism, effectively captures relational heterogeneity, local entropy, and feature-level disparities in the graph. Extensive evaluations show that NeighborSense achieves consistently superior performance compared to state-of-the-art GNN-based bot detection methods.

## Related work

Existing approaches to social bot detection can be primarily grouped into three categories: feature-based methods, graph-based methods, and deep learning-based approaches.

**Feature-based bot detection methods** represent one of the earliest strands of research in identifying social bots. These approaches typically rely on features extracted from user profiles, posted content, and behavioral traces, which are analyzed using a variety of techniques—from heuristic filtering mechanisms such as blacklisting and URL blocking to supervised discriminative models. As a result, bot detection within this category is commonly framed as a supervised classification problem.

A well-known instance is Botometer [2], which utilizes over 1,000 attributes organized into six categories: user profile, social connections, network structure, temporal dynamics, content and language use, and sentiment. The system employs a random forest model developed from a large corpus of annotated data. In a different vein, Cresci *et al*. [8] proposed a “digital DNA” encoding scheme to represent user activity sequences, with bot clusters identified through longest common substring analysis. Yang *et al*. [9] addressed generalization and scalability issues in contemporary detection systems by introducing a lightweight random forest model that operates on minimal user metadata, allowing real-time processing of large-scale tweet streams. A growing number of recent studies have incorporated natural language processing techniques to extract semantic information from user-generated textual content. For example, Kudugunta *et al*. [10] and Wei *et al*. [11] adopted recurrent architectures utilizing both standard and bidirectional LSTM networks to capture tweet semantics for classification purposes.Similarly, BGSRD [12] introduced a hybrid framework that synergizes BERT and Graph Convolutional Networks (GCNs) to enhance tweet representation through feature-level fusion.

**Graph-based methods** for detecting bots mainly include techniques utilizing random walks (RW) and those employing loopy belief propagation (LBP). Both paradigms operate within a semi-supervised learning framework, propagating labels from a subset of annotated nodes to infer the labels of unknown nodes. RW-based methods are based on the assumption that random walks originating from legitimate users are more likely to traverse within legitimate regions of the graph, whereas Sybil nodes are less likely to reach legitimate nodes within a short walk length. Inspired by this principle, early systems such as SybilGuard [13] and SybilLimit [14] spurred the development of numerous extensions, including SmartWalk [15], SybilInfer [16], SybilRank [17], SybilWalk [18], and Integro [19]. More recently, Zhang *et al*. [20] introduced a Social and Activity Network (SAN)—a two-layer hypergraph integrating user friendships and activities—and proposed SybilSAN, which couples three RW-based algorithms for enhanced Sybil detection. In contrast, LBP-based methods model the joint label distribution across the graph using Markov random fields and employ belief propagation to iteratively approximate posterior probabilities for unlabeled nodes. Originating from the influential SybilBelief [3], several improvements have been made to enhance scalability and robustness. SybilSCAR [4] integrates concepts from both RW and LBP traditions, streamlining the update rules for more efficient posterior estimation. SybilFuse [21] incorporates local structural attributes via pre-trained classifiers to refine node priors before applying LBP. GANG [22] adapts LBP to directed graphs through customized edge potential functions and offers a scalable matrix-based implementation. SybilEDGE [23] leverages unique graph structures derived from “friend request-response” interactions rather than static friendships, facilitating rapid detection of fake accounts through internal platform data. Nevertheless, graph-based detection systems remain susceptible to adversarial manipulations that alter graph topology to evade detection [24].

Recent works with **graph representation learning or GNN-based models** have advanced social bot detection by jointly modeling user attributes and graph structural information. TrustGCN [25] incorporates graph neural networks with social graph-based defense mechanisms on a “friend request” graph to improve resilience against adversarial attacks. Bot2vec [26] extends Node2vec by integrating community detection with graph representation learning for enhanced bot identification. BotRGCN [5] employs relational graph convolutional networks on Twitter follower networks, integrating multi-modal user semantics, properties, and neighborhood features. SATAR [27] proposes a self-supervised learning framework that leverages tweets, metadata, and neighborhood context to learn user representations. Feng *et al*. [6] proposed a heterogeneity-aware framework that incorporates heterogeneous information networks (HINs) and utilizes relational graph transformers enhanced with semantic attention. Their method, especially in conjunction with BotRGCN, attains top-tier results on the TwiBot-20 benchmark. More recently, BotCL [28] applies graph contrastive learning with data augmentation to obtain more robust node representations. To capture decentralized group interactions, Liu *et al*. [29] propose AMGP, an adaptive multiscale hypergraph model for modeling complex relational structures. Beyond bot detection, recent studies in graph representation learning emphasize exploiting higher-order structural information for improved graph understanding, e.g., incorporating graph-level/node-level mutual information and structural constraints for graph clustering [30]. While these methods target clustering rather than bot detection, they highlight the importance of capturing structural patterns beyond immediate neighbors. In contrast, our work focuses on node-level neighborhood statistics and uses them to modulate relation-wise aggregation in a supervised bot detection setting.

## Methodology

**Problem formulation:** Let G=(V,E) be a *direction-aware* multi-relational social graph, where *V* is the set of users and *E* is the set of *typed directed* edges. Each edge is represented as a triple (*u*,*r*,*v*), indicating that user *u* has relation type *r toward* user *v*, with r∈R.

**Direction-aware relations.** Although our backbone uses the standard relation-wise aggregation form, we explicitly encode edge directions as distinct relation types. For each relation r∈R, we introduce its reverse relation *r*^−1^ (e.g., *follow* vs. *has_follower*) and include both in the relation set. Concretely, for every observed edge (*u*,*r*,*v*), we additionally add the reverse typed edge (*v*,*r*^−1^,*u*). Hence, *E* contains edges of both original and reverse relations, enabling direction-aware message passing.

For node *i* and relation *r*, we define the relation-specific neighborhood on *incoming* edges as

$$N_{i}^{r}=\{\;j|(j,r,i)\in E\;\}.$$

With this definition, the aggregation in Eq 2 is direction-aware because the semantics of “incoming vs. outgoing” are encoded by different relation types (*r* and *r*^−1^), each having its own parameters and gate.

**Fig 2:**
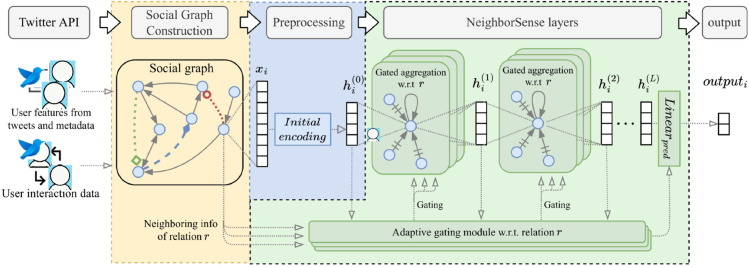
Overview of the proposed NeighborSense framework.

**Overall framework:** Fig 2 illustrates the overall architecture of the proposed NeighborSense. The model begins by constructing a multi-relational social graph from user interaction data and features derived via the Twitter API, including tweet content and profile information. For each node, a preliminary encoding of the node’s attributes is performed during preprocessing as shown in the yellow and blue areas in the figure.

The green area of Fig 2 starts from an R-GCN as our backbone relational heterogeneity modeling. Then, for the subgraph of each relation, we apply the proposed NeighborSense layer to perform gated aggregation per node, where the aggregation strength is determined by neighborhood information (e.g., labels and features) from the previous layer.

In this process, we maintain a cross-layer adaptive gating module, which receives the node representations from the previous layer and adapts the aggregation strength for the center node based on the pre-classification results.

Finally, we classify the last hidden representation as bot and human using the same linear classifier as in pre-classification, and train the model parameters.

### Social graph construction and data preprocessing

Social interactions are inherently directional. To preserve direction information in relational message passing, we model each direction as a distinct relation type. Specifically, for each observed directed relation *r* (e.g., *follow*), we introduce a reverse (inverse) relation *r*^−1^ (e.g., *has_follower*). For every edge (*u*,*r*,*v*) extracted from the raw data, we add the corresponding reverse edge (*v*,*r*^−1^,*u*). As a result, the constructed graph contains a set of *direction-aware* relation types, and message passing can capture different semantics carried by opposite directions.

User features, extracted from both tweets and metadata, are encoded following the procedure established in [5, 6]. Specifically, in the preprocessing stage, the node information is fed into a linear layer by type for encoding and then concatenated to obtain a low-dimensional dense representation of each user:

$$x_{i}^{\left(0\right)}={\mathrm{concat}}_{k}\left(\sigma \left(W_{k}\cdot x_{i,k}+b_{k}\right)\right),$$ (1)

where *x*_*i*,*k*_ is the *k*-th type of original feature (possibly of high dimensionality) of user *i*, *W*_*k*_ and *b*_*k*_ denote learnable parameters for encoding *k*-th type feature, and *σ* is the relu activation function.

Next, the preprocessed node representations are fed into the NeighborSense layers, as shown in the green area in Fig 2, which consists of an *adaptive gating module* attached to a *backbone R-GCN*.

### Backbone R-GCN layer

The update function of NeighborSense for a single layer *l* is as follows:

$$h_{i}^{\left(l+1\right)}=\sigma \left(W_{0}^{\left(l\right)}h_{i}^{\left(l\right)}+\sum_{r\in R}z_{i,r}\sum_{j\in N_{i}^{r}}\frac{1}{\left|N_{i}^{r}\right|}W_{r}^{\left(l\right)}h_{j}^{\left(l\right)}\right),$$ (2)

where $N_{i}^{r}$ denotes the *in-neighborhood* of node *i* under relation *r*, i.e., $N_{i}^{r}=\{j|(j,r,i)\in E\}$. Note that *R* includes both original and reverse relations (e.g., *follow* and *has_follower*), so Eq 2 is direction-aware: messages along opposite directions are modeled as different relation types with their own parameters and gates.

### Adaptive gating module

Next, we propose two intuitive metrics to capture neighborhood distribution and heterogeneity, and use them to calculate the aggregation gate *z*_*i*,*r*_.

**Why entropy and PMI-inspired similarity.** We choose entropy and a PMI-inspired similarity as the core neighborhood statistics for three reasons. First, they are *principled and complementary*: entropy measures the dispersion of the local bot/human distribution under each relation, capturing how mixed or uncertain the neighborhood is, while the PMI-inspired similarity measures (prior-normalized) agreement between the central node and its neighbors, capturing relation-dependent homophily/heterophily. Second, they are *lightweight and scalable*: both statistics can be computed from relation-wise neighborhood label proportions, avoiding expensive pairwise feature similarities and remaining feasible on large graphs. Third, they are *aligned with our objective*: bot detection is highly imbalanced and relation semantics vary; prior-normalized agreement helps distinguish non-trivial neighborhood consistency from agreement that arises simply due to class imbalance.

For local entropy awareness, the key idea is to estimate the diversity of a particular node’s neighborhood. For node *i*, we compute the entropy among its neighbors:

$$\begin{array}{cc} Etp(i,r) & =-p_{h,r}(i)\log p_{h,r}(i)-p_{b,r}(i)\log p_{b,r}(i),\;\\ p_{h,r}(i) & =\frac{\left|\{j\in N_{i}^{r}|y_{j}=human\}\right|}{\left|N_{i}^{r}\right|},\;\\ p_{b,r}(i) & =\frac{\left|\{j\in N_{i}^{r}\}|y_{j}=bot\right|}{\left|N_{i}^{r}\right|}, \end{array}$$ (3)

where *p*_*h*,*r*_(*i*) and *p*_*b*,*r*_(*i*) are the proportions of humans and bots in the relation-*r* neighborhood. Eqs 3 and (4) are written with discrete labels for clarity; in practice, these neighborhood statistics are estimated from the shortcut-classifier outputs of the previous layer (i.e., soft probabilities), and we apply a small smoothing constant *ε* when needed to avoid numerical issues when a probability/count is close to zero. A larger *Etp*(*i*,*r*) means that the label distribution of user *i*’s neighborhood is more dispersed with respect to relation *r*.

To quantify local label/semantic heterogeneity, we adopt a *PMI-inspired* similarity that measures whether node *i* and its relation-*r* neighborhood exhibit stronger-than-expected label agreement.

**PMI-based intuition.** Let

$$p_{i,r}^{\text{loc}}=\frac{\left|\left\{\;j\in N_{i}^{r}|y_{j}=y_{i}\;\right\}\right|}{\left|N_{i}^{r}\right|}$$

denote the local probability that a relation-*r* neighbor shares the same label as node *i*, and let

$$p_{i}^{\text{glob}}=\frac{\left|\left\{\;j\in V|y_{j}=y_{i}\;\right\}\right|}{\left|V\right|}$$

be the global prior probability of observing label *y*_*i*_.

The classical pointwise mutual information (PMI) between the events “a relation-*r* neighbor has label *y*_*i*_” and “node *i* has label *y*_*i*_” can be written as

$$PMI(i,r)=\log\frac{p_{i,r}^{\text{loc}}}{p_{i}^{\text{glob}}}.$$

Since the gating module only requires a *monotonic* measure of relative agreement strength, we use the log-free surrogate $\exp(PMI(i,r))=\frac{p_{i,r}^{\text{loc}}}{p_{i}^{\text{glob}}}$. Dropping the graph-level constant factor |V| (which does not affect ranking across nodes/relations within the same graph) yields the following computationally efficient score, which we refer to as a *PMI-inspired similarity*:

$$Sim(i,r)=\frac{\left|\left\{\;j\in N_{i}^{r}|y_{j}=y_{i}\;\right\}\right|}{|N_{i}^{r}|\cdot \left|\left\{\;j\in V|y_{j}=y_{i}\;\right\}\right|}.$$ (4)

In practice, to handle unlabeled users and reduce noise in early layers, we compute the above quantities using the shortcut classifier outputs from the previous layer (i.e., pseudo-labels or soft probabilities), and we apply a small smoothing constant *ε* to avoid numerical issues when a count is close to zero. A larger *Sim*(*i*,*r*) indicates stronger (prior-normalized) agreement between node *i* and its relation-*r* neighborhood (i.e., higher homophily under relation *r*). In this scenario, it has been shown that when neighborhood agreement is very high, aggregation may bring limited gains, and representations before and after aggregation can be nearly the same [31].

**Discussion of alternative neighborhood indicators.** We acknowledge that other neighborhood indicators could also be used to quantify dispersion and agreement. For neighborhood dispersion, typical alternatives include *Gini impurity* ($1\;-\;\sum_{c}p_{c}^{2}$) and variance-based measures, which are closely related to entropy and capture similar “mixedness” trends in the binary (bot/human) setting. We choose entropy for its information-theoretic interpretation and smooth sensitivity to changes in the label proportions. For neighborhood agreement, a natural alternative is the *plain homophily ratio* $\frac{\left|\{j\in N_{i}^{r}|y_{j}=y_{i}\}\right|}{\left|N_{i}^{r}\right|}$, or feature-based similarities (e.g., cosine similarity). However, without prior normalization, the plain homophily ratio can be biased under class imbalance, while feature-based similarities introduce additional computation and may be less stable across relation types. Our PMI-inspired score explicitly normalizes local agreement by the global label prior, providing a lightweight and more comparable signal across relations and datasets. A systematic empirical comparison of a broader set of neighborhood indicators is an interesting direction for future work; in this revision, we focus on clarifying the motivation and properties of entropy and PMI-inspired similarity that underpin our gating design.

**Effect of imperfect predictions on message propagation.** We note that the neighborhood statistics in Eqs 3–(4) depend on the previous-layer pre-classification. This does not lead to uncontrolled error propagation for the following reasons. First, in implementation we compute these statistics using *soft* shortcut-classifier outputs (probabilities) rather than hard pseudo-label counting, so uncertain predictions contribute less and the estimates are more stable in early layers. Second, the statistics are neighborhood-averaged; hence the influence of any individual mistake is attenuated by $1/|N_{i}^{r}|$. Third, the gate is bounded by the sigmoid function ($z_{i,r}\in (0,1)$) and standardized by Norm(·), which prevents unbounded amplification and keeps the gate comparable across layers. Finally, even when *z*_*i*,*r*_ becomes small for a noisy relation, the backbone update in Eq 2 still preserves node *i*’s self-information through the self-feature term, so the representation is not solely determined by potentially noisy neighbor messages. The gate *z*_*i*,*r*_ is computed from *Etp* and *Sim* using the following equation:

$$z_{i,r}=\;\sigma (\eta_{r,1}\cdot Norm(Sim(i,r))\;+\eta_{r,2}\cdot Norm(Etp(i,r))).$$ (5)

For each node, gate *z*_*i*,*r*_ is obtained by the joint action of *Etp* and *Sim* and is controlled by two parameters $\eta_{r,1}$ and $\eta_{r,2}$. The activation function *σ* limits the value of *z*_*i*,*r*_ to (0,1). *Norm* is the layer normalization operation to make them comparable across layers.

**When *z***_***i*,*r***_ **approaches 0.** When *z*_*i*,*r*_ becomes small, NeighborSense intentionally suppresses messages from relation *r* for node *i* to avoid injecting noisy or irrelevant neighborhood information under that relation. This does *not* remove the effective information of node *i*. First, Eq 2 always contains the self-feature term $W_{0}^{\left(l\right)}h_{i}^{\left(l\right)}$, so the representation of node *i* preserves its own information even if one relation is strongly down-weighted. Second, the gate is *node-wise and relation-wise*: setting $z_{i,r}\approx 0$ for a specific relation only filters that relation, while aggregation from other relations can still contribute. Third, since *z*_*i*,*r*_ is bounded by the sigmoid function ($z_{i,r}\in (0,1)$), it does not create a hard disconnection; instead, it performs soft, selective filtering of relation-specific neighbor messages. Therefore, the model avoids losing the node’s effective information while preventing harmful aggregation. Here we do not follow the design in [31], which argues that larger local entropy implies a warning that the homophily effect may not hold, and the aggregated information may be noise, and larger center-neighbor node similarity implies further aggregation is unnecessary. However, in the case of different relations, we actually do not know whether the aggregation strength should be larger or smaller when *Etp* (or *Sim*) is larger. For example, the blacklist relation shown in Fig 1(c) embodies inherent heterogeneity, a larger *Sim* does not mean that aggregation is unnecessary. We therefore devise a more loose gating calculation, where the interaction of *Etp* and *Sim* are linearly combined with the parameters. By doing so, the monotonicity between *z*_*i*,*r*_ and *Etp* (or *Sim*) is determined by the parameters, adapting it to more diverse aggregation patterns.

**Fig 3:**
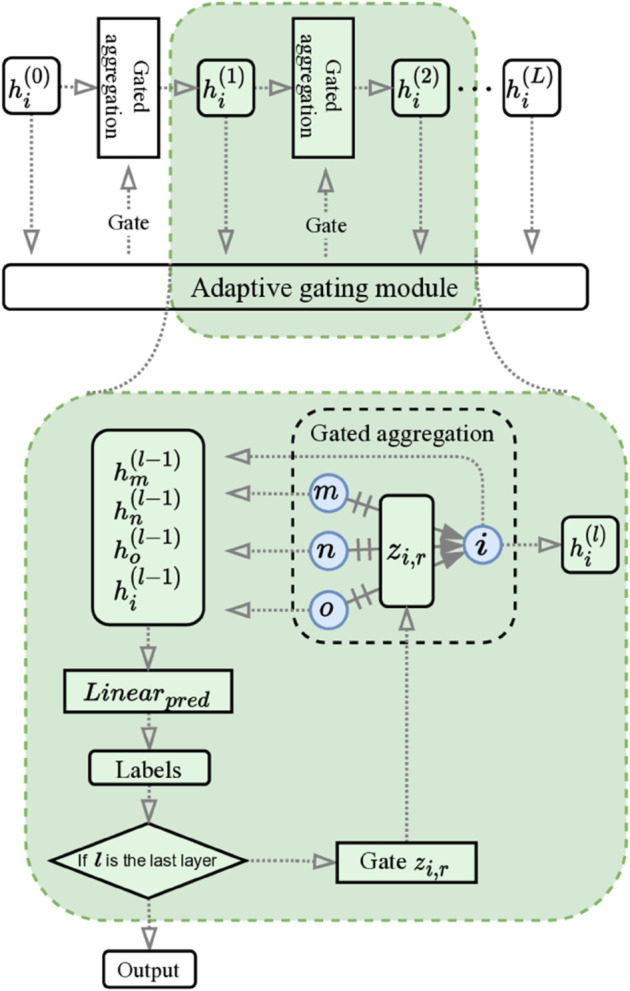
The NeighborSense layer with the adaptive gating module. The dashed box illustrates how the gate *z*_*i*,*r*_ is computed from neighborhood statistics and used to control relation-wise aggregation.

As shown in Fig 3, we integrate the proposed gate *z*_*i*,*r*_ by an adaptive gating module. It resembles a shortcut (skip-connection), as we use a linear layer *Linear*_*pred*_ as follows to project every hidden layer representation $h_{i}^{\left(l-1\right)}$ to a prior predicted label *y*_*i*_, with which we calculate *l*-th layer’s gate *z*_*i*,*r*_ as well as the final prediction:

$$y_{i}=Softmax(W_{pred}h_{i}^{\left(l-1\right)}+b_{pred}),$$ (6)

where $W_{pred}h_{i}^{\left(l-1\right)}$ and *b*_*pred*_ are the learnable parameters.

### Output and loss function

Finally, from the last hidden representation of the above backbone network $h_{i}^{\left(L\right)}$, we obtain the classification result ${\hat{y}}_{i}$ by linear layer *Linear*_*pred*_ as follows:

$${\hat{y}}_{i}=Softmax(W_{pred}h_{i}^{\left(L\right)}+b_{pred}).$$ (7)

The model is optimized using the binary cross-entropy loss, which is defined as follows:

$$Loss=-\sum_{i\in V_{L}}({\bar{y}}_{i}\log{\hat{y}}_{i}+(1-{\bar{y}}_{i})\log\left(1-{\hat{y}}_{i}\right)).$$ (8)

where ${\hat{y}}_{i}$ is the model prediction and ${\bar{y}}_{i}$ is the ground truth.

## Experiment

### Experiment settings

**Dataset:** Our proposed detection model leverages graph structures by incorporating both user attributes and overall graph topology. We evaluate our approach on two publicly available benchmarks for Twitter bot detection: TwiBot-20 [32] and TwiBot-22 [33], both of which provide multimodal user data and relational information.

To ensure fair and consistent comparison with existing studies, we adopt the standard data partitions provided by these benchmarks. The detailed statistics of both datasets are summarized in Table 1.

**Table 1:** Dataset statistics.

Datasets	users	edges	Bots	human	train/validate/test
TwiBot-20	229,573	455,958	5,273	6,589	70%/20%/10%
TwiBot-22	1,000,000	6,818,501	139,943	860,057	70%/20%/10%

**Note:** #Users denotes the total number of nodes in the constructed graph (including both labeled and unlabeled accounts), whereas #Bots and #Human denote the numbers of *labeled* bot/human accounts in the benchmark split (i.e., within $V_{L}$). Therefore, in general, #Bots + #Human = $\left|V_{L}\right|$ and #Users = $\left|V_{L}|+|V_{U}\right|$, so #Bots + #Human may be smaller than #Users.

In terms of social relations, Twibot-20 contains only “follow" and “followed by" relations between users; while Twibot-22 has 14 relations, in addition to “follow" and “followed by", we also convert “retweeted", “like", “quoted", “replied", “mentioned" into relations between users. Further, we also reverse-represent these 5 relations and eventually build an inter-user information network with 12 relations as Table 2 shows.

**Table 2:** Relations between users extracted from Twibot-22.

Relation	Reversed relation	Description	Number
follow	followed by	user A follows user B	3743634
retweet	retweeted by	user A retweets user B’s tweet	1580643
like	liked by	user A likes user B’s tweet	595794
quoted	quoted by	user A quoted user B’s tweet	289476
replied	replied by	user A replied user B’s tweet	1114981
mentioned	mentioned by	user A mentioned user B’s tweet	4759388

**Comparison baselines:** The proposed NeighborSense is evaluated against the following methods:

- Botometer [2] utilizes over 1,000 user attributes for bot identification.
- Yang *et al*. [9] employ a random forest classifier using limited user metadata and engineered features.
- Wei *et al*. [11] apply recurrent neural networks to model user tweets for classification.
- Kudugunta *et al*. [10] integrate both tweet semantics and user metadata for detection.
- SATAR [27] is a self-supervised framework that learns user representations from tweets, metadata, and local neighborhood information.
- BotRGCN [5] uses relational graph convolutional networks to learn user representations and detect bots.
- Feng *et al*. [6] introduce a heterogeneity-aware graph method that constructs heterogeneous information networks (HINs) and employs relational graph transformers with semantic attention mechanisms. Together with BotRGCN, this method achieves state-of-the-art results on the TwiBot-20 benchmark.

**Evaluation metrics:** The performance of bot detection methods is assessed using the following metrics:

- Accuracy is the most common evaluation metric. However, the unbalanced distribution of the dataset (there are far fewer labeled bot accounts than labeled human accounts in TwiBot-20) leads to a greater impact on the results in terms of the correctness of the classification of humans under this metric.
- For F1-score, we report the class-wise performance for the bot class, which is more informative under class imbalance. When the classes are highly imbalanced, F1 can better reflect minority-class performance than accuracy.
- MCC treats the true labels and predicted labels as two binary random variables and measures the correlation between them. Its value falls within the range of –1 to 1, with a value of 0 suggesting that the classifier performs at a level equivalent to random chance.

**Implementation:** We implement the proposed Twitter bot detection framework using PyTorch, Deep Graph Library (DGL) [34], and the Hugging Face Transformers library [35]. The hyperparameter configurations used in our experiments are provided in Table 3. Unless otherwise stated, we initialize the relation-specific gating parameters $\eta_{r,1}$ and $\eta_{r,2}$ to 0 for all relation types, so that the initial gate value is around σ(0)=0.5, and learn them end-to-end during training.

**Table 3:** Parameter settings.

Parameter	Value
Number of NeighborSense layers	3
Optimizer	AdamW
Batch size	Consistent with data set size
Epochs	100
Learning rate	0.001
Dropout rate	0.3
Embedding size	128

### Performance results

As summarized in Table 4, we compare the performance of various state-of-the-art bot detection methods on the Twibot-22 and Twibot-20 datasets. The symbol *graph-based* indicates whether a method incorporates user interaction graphs, with the number of relation types denoted in parentheses. Similarly, the annotation *deep* indicates the employment of deep learning techniques. When applicable, the approximate number of parameters is provided within parentheses. Overall, the results indicate that methods incorporating relational information consistently outperform those relying solely on node attributes, underscoring the importance of graph structural data in Twitter bot detection. Furthermore, the proposed method achieves the best performance on Twibot-22 when leveraging the most diverse set of social relations, demonstrating its ability to effectively model heterogeneous relational data. It is also observed that deep learning-based models generally attain better detection performance at the cost of increased model size. Despite this, the proposed method, equipped with multiple layers, outperforms competing approaches with larger model capacities. Notably, it maintains a significant advantage even when using only two relation types, matching the input complexity of other GNN baselines.

**Table 4:** Benchmarking results of bot detection approaches.

Method	TwiBot-20			TwiBot-22			Relations	Deep
	Acc	F1	MCC	Acc	F1	MCC		
Botometer [2]	0.558	0.489	0.155	0.498	0.425	0.132	–	No
Yang *et al*. [9]	0.819	0.854	0.664	0.739	0.275	0.425	–	No
Kud *et al*. [10]	0.817	0.751	0.671	0.658	0.516	0.217	–	Yes
Wei *et al*. [11]	0.712	0.753	0.419	0.702	0.536	0.299	–	Yes
SATAR [27]	0.841	0.864	0.686	0.787	0.571	0.383	–	Yes
BotRGCN [5]	0.846	0.870	0.702	0.796	0.575	0.432	2	Yes
Feng *et al*. [6]	0.866	0.882	0.714	–	–	–	2	Yes
NeighborSense	0.8690	0.8834	0.7370	0.807	0.545	0.439	2	Yes
	–	–	–	0.817	0.561	0.450	12	Yes

**Table note:** The experimental results were obtained from the official TwiBot-22 repository [36].

**Fig 4:**
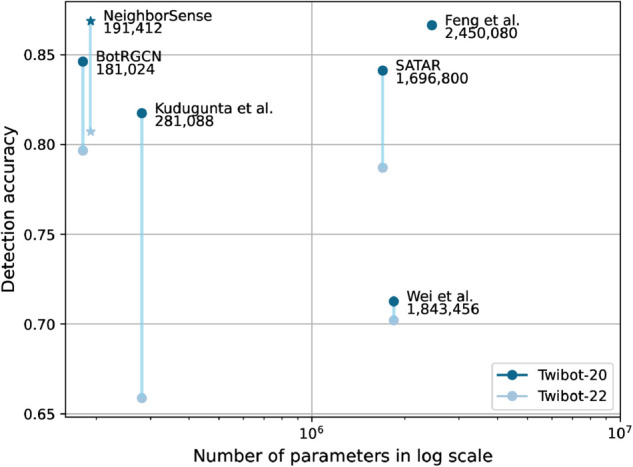
Model Scale vs. Detection accuracy.

We have estimated the parameter counts of several recent deep learning-based bot detection approaches. It should be noted that, as some model configurations were not fully specified in their original publications, parameter quantities were inferred based on commonly adopted architectural choices. Fig 4 depicts the number of trainable parameters (on a logarithmic scale) versus detection accuracy across two benchmark datasets, Twibot-22 and Twibot-20, evaluated on multiple state-of-the-art deep learning models. This comparison offers an approximate measure of model complexity and computational requirements. As shown, the proposed NeighborSense attains competitive performance even under constrained model size, demonstrating favorable efficiency among compared methods.

**Fig 5:**
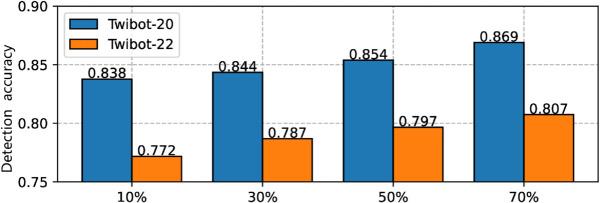
Model performance with training data proportion ranging from 10% to 70% of the whole dataset.

As a supervised approach dependent on labeled examples, it is essential to evaluate the model’s generalization capability and data efficiency under limited annotation. Fig 5 illustrates the performance when varying proportions (from 10% to 70%) of the full dataset are used as training samples. The results indicate that our method maintains satisfactory detection accuracy even when the amount of labeled training data is reduced to only 10%.

### Aggregation analysis

In this subsection, we examine the adaptive gating mechanism and analyze how it works in practice.

**Fig 6:**
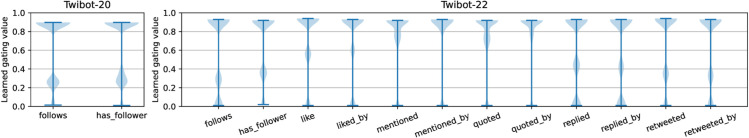
Distribution of *z*_*i*,*r*_ for different relations.

First, we open up our model and examine how the gates control the aggregation strength of each node. We count the distribution of node-wise aggregation gate *z*_*i*,*r*_ over the graph as shown in Fig 6.

It can be seen that the two relations “follow" and “has_follower" in the Twibot-20 dataset correspond to the two directions of the “follow" relation, respectively. The aggregation strength of most nodes is concentrated around 0.9 to 1, and we expect that aggregating over these nodes will enhance performance. A subset of nodes has aggregation strength close to 0. The composition of *z*_*i*,*r*_ shows that this may be due to local heterogeneity or local entropy that makes the current node unsuitable for aggregation, such as when human points are followed by a large number of bots. Another subset has gate values around 0.3, resulting from the mutual compromise of local heterogeneity or local entropy interaction. The 12 relationships in the Twibot-22 dataset also show similar distribution characteristics, and the differences in the distribution of different relationships are mainly reflected in the aggregation strength of those “moderate" nodes.

If we turn off the adaptive gating mechanism, i.e., fix all gating values to 1, the over-smoothed aggregation will reduce the detection accuracy of our model to 75.59% and 72.21% for Twibot-20 and Twibot-22, respectively.

**Fig 7:**
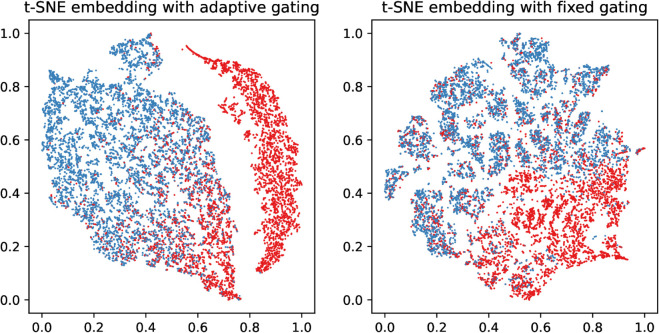
Visualization of human and bot representations on Twibot-20 via t-SNE 2-D projections.

As shown in Fig 7, we perform a t-SNE 2D projection of the feature representation using the adaptive gating mechanism and the one using fixed aggregation strength, and we can see that the node representation using the adaptive gating mechanism has more significant differentiability, while the node representation using fixed aggregation strength appears more blending, which may be caused by the nodes being over-smoothed during aggregation.

## Conclusion

In this study, we examine local interactive behaviors between bots and human users and introduce two novel metrics derived from neighborhood statistics. Based on these metrics, we propose NeighborSense, a social bot detection method that incorporates gated aggregation to adaptively integrate multi-relational information for different users. The proposed method effectively captures relational heterogeneity, local entropy, and feature distribution variations within the graph. Experimental evaluations demonstrate that NeighborSense delivers robust and superior detection accuracy compared to state-of-the-art approaches.
